# Age-Related Differences in Postural Balance Among Adults Aged 50–80 Years: An Observational Study

**DOI:** 10.3390/jfmk11030277

**Published:** 2026-07-18

**Authors:** Gloria Amalfi Luna-Corrales, Maria Camila Rangel-Piñeros, Carol Cardozo-Guzmán, Ingrid Tolosa-Guzman, María Constanza Trillos-Chacón, Martha-Rocío Torres-Narváez

**Affiliations:** 1 Rehabilitation Science Research Group, School of Medicine and Health Sciences, Universidad del Rosario, Bogotá 111221, Colombia; gloria.luna@urosario.edu.co (G.A.L.-C.); ingrid.tolosa@urosario.edu.co (I.T.-G.); martha.torres@urosario.edu.co (M.-R.T.-N.); 2 Research and Production Group, Instituto Nacional de Salud, Bogotá 111221, Colombia; mariaca.rangel@urosario.edu.co; 3 Independent Researcher, Bogotá 111221, Colombia; carol.cardozo@urosario.edu.co

**Keywords:** postural balance, adult, cross-sectional study, public health

## Abstract

Global ageing has increased the population of people aged over 60 years, especially in low- and middle-income countries. **Objective:** To evaluate the postural control of adults aged 50–80 years and its relationship with age. **Methodology:** We conducted a cross-sectional observational study in Bogotá and nearby municipalities with 211 participants aged 50 to 80 years. We used the Balance Evaluation Systems Test (BESTest) to measure balance and excluded individuals with mobility-related conditions. We used statistical tests to analyse data from the three age groups. **Results:** Significant differences in postural control were found between age groups; a progressive decline in balance was shown, especially in those over 70 years old. More women than men participated in the study; performance on tasks such as standing on one leg and mobility-related activities decreased with age. **Conclusions:** Balance performance was lower in older age groups, suggesting an age-related decline in postural control. The findings suggest that postural balance performance is lower in older age groups, especially after 70 years of age. These results support the importance of early preventive exercise programmes aimed at maintaining balance and reducing fall risk.

## 1. Introduction

Ageing of the global population is accelerating; global life expectancy now exceeds 70 years, thereby arousing interest in the study of longevity. The World Health Organisation (WHO) estimates that by 2030, one in six individuals will be over 60 years old (around 1 billion) by 2030. Height and weight decrease with age, especially after age 70 [1], with such difference being greater in males [2]. Ageing is accompanied by gradual changes in the musculoskeletal, sensory and neuromotor systems that contribute to postural control. Although these changes do not occur abruptly at a specific age, subtle alterations may emerge during middle adulthood and become more pronounced with advanced age. More advanced ageing is also associated with physiological changes that may compromise the body’s ability to respond to postural disturbances, coexisting with hearing loss, musculoskeletal pain, chronic diseases, and geriatric syndromes such as frailty and dementia, all of which represent major global public health challenges [3].

Colombia’s National Statistics’ Administrative Department (DANE) stated that 51.2% of the country’s total population in 2024 (52,695,952 people) were women [4]; the country’s total population has been estimated to be 54,705,567 in 2030, an increase of 3.7%. Old age is associated with less social participation and increased disease and disability in Colombia and possibly throughout Latin America, thereby limiting the prospects for active and healthy ageing. The Active Ageing Index (AAI) in Colombia for 2020 was 37.4% [5].

Ageing is a complex, continuous, and multifaceted process that involves ongoing biological, psychological, and social changes throughout life [6,7]. It involves both the physical challenges arising from molecular and cellular deterioration and the transitions and adaptations to life’s new stages and social roles, thereby evidencing great variability regarding the way each person ages [6]. Various pathologies appear during such a process, which can increase the risk of falling and decrease functionality and independence [8,9].

Balance is a protective factor concerning adult functionality [10,11] (i.e., people’s ability to perform basic daily activities, thereby enabling them to survive, take care of themselves, preserve their independence, and form part of a home and a community); thus, validated instruments are required for assessing balance in older people, along with the pertinent training for professionals regarding its study and analysis, as well as compiling accurate information about this motor skill’s behaviour in the ageing population [7,8,9].

Balance or postural control plays a fundamental role in performing activities of daily living. Static balance involves the ability to maintain a posture, whereas dynamic balance provides stability for body movement [10,11,12,13,14,15,16]. Understanding the functional profile of the ageing population helps structure promotion, prevention, and intervention programmes for a full and active life. This study aimed to evaluate postural control in adults during ageing.

## 2. Materials and Methods

We conducted a cross-sectional observational study. We selected a convenience sample of 211 participants from Bogotá, Colombia, who voluntarily enrolled in the study. All participants signed an informed consent form, answered questions about their health history, and completed a BESTest from Oregon, United States, regarding postural control system assessment. For the purposes of this article, the postural control performance items that provided relevant quantitative data on time (seconds) and distance (centimetres) were selected. The Research Ethics Committee of Universidad del Rosario approved this study (DVO005-1-170-CEI698). We included participants aged 50 to 80 years who could follow verbal instructions and walk 6 m without assistance. We excluded those who had been hospitalised during the previous 6 months; had cognitive, sensory, or vestibular deficits; or had neuromuscular or cardiopulmonary conditions affecting movement or stability. Two physiotherapists with more than 10 years of experience administered the BESTest to all participants in a Bogotá hospital over a 36-month period (from February 2017 to February 2020) [10]. We followed a previously standardised procedure that involved evaluating the following nine tasks: (1) functional reach, (2) sitting down on the floor and getting up, (3) standing on one leg (left and right), (4) stepping up onto a step, alternately, (5) sensory integration (best performance), (6) walking on a stable surface (gait), (7) overcoming obstacles, (8) timed up-and-go, and (9) get-up-and-go with dual task, measuring execution time [16].

We stored the data in an Excel database and protected participants’ identities using an alphanumeric code. We conducted the study in accordance with Good Clinical Practice and data protection regulations.

For the statistical analysis, we created three age groups: group 1 (≥50 and ≤60 years), group 2 (>60 and ≤70 years), and group 3 (>70 and ≤80 years). We performed a bivariate analysis by age group to describe the sample characteristics. We presented absolute and relative frequencies and compared *p*-values using the chi-square test for homogeneity.

We conducted a frequency analysis for sensory orientation and test execution time. We reported the mean and standard deviation for continuous variables, whereas we used the median and interquartile range (IQR) for skewed data or data with outliers. We used the Shapiro–Wilk test to assess the normality of the distribution of the analysed items.

We used the Mann–Whitney U test to compare differences by sex. After a significant Kruskal–Wallis test, we applied post hoc pairwise comparisons to identify specific group pairs with statistically significant differences in non-parametric data. We used the chi-square test to identify differences between age groups and applied the Dunn–Bonferroni test to adjust for multiple comparisons. We set the significance level at *p* = 0.05.

## 3. Results

The study participants were mostly healthy females (64.2–71.3%) at the time of evaluation (64.9–73.9%) (Table 1).

We observed a body mass index (BMI) range of 18.3–44.6 kg/m^2^ among all participants, with a median of 27.4 kg/m^2^. BMI showed a similar distribution across all age groups (*p* = 0.4894). Regarding stature, the median for all participants was 1.56 m, and we recorded significant differences among the groups [Table 1].

Table 2 shows the overall bivariate analysis results by sex. The Mann–Whitney test showed significant sex-based differences in performance on the functional reach test, walking on a stable surface, and obstacle clearance tests.

We found minimal variability in stability time in the bipedal position. All participants (100%; *n* = 211) maintained equilibrium for 30 s with their eyes open on a stable surface. In addition, 99.5% maintained equilibrium with their eyes closed on a stable surface and with their eyes open on an unstable surface, whereas 79.6% maintained equilibrium with their eyes closed on an unstable surface. Table 3 shows the results of bivariate analysis by age group. All participants took a mean of 8.1 s to sit down on the floor and then stand up, with times ranging from 0 to 75 s. Participants achieved a median functional reach test (FRT) score of 25.8 cm, with values ranging from 3.5 to 43 cm. In the one-legged standing test, participants maintained the position for a mean of 10.3 s on the right leg and 9.8 s on the left leg, with both sides showing a range of 0 to 30 s. Participants took a median of 8.9 s to step up onto a step, with times ranging from 1.6 to 23.2 s. Group 1 had better and greater reach times, indicating better performance with the right leg (Table 3).

Participants took a median of 6.4 s to walk on a stable surface over 6 m, with times ranging from 3.3 to 29.8 s. In the gait stability test with obstacle clearance over 6 m, participants took a median of 6.8 s, with times ranging from 3.3 to 13.8 s. Participants completed the timed up-and-go (TUG) test over 3 m in a median of 9.1 s, with times ranging from 5.8 to 15.1 s, whereas they completed the dual-task TUG in a median of 9.9 s, with times ranging from 6.1 to 23.4 s. Group 3 had the longest times regarding such tests (Table 3).

Overall, 13 of the 211 participants were unable to perform the sit-up and stand-up tests. In addition, 51 participants could not perform the one-legged standing test: 24 on the right leg and 27 on the left leg. Some participants had a 0.0 minimum interval on the aforementioned tests, meaning that such people could not do them (Table 3).

The Kruskal–Wallis test rejected the null hypothesis for all BESTest items, indicating that performance on postural control tasks differed among age groups (Table 3).

We also performed a post hoc analysis using the Dunn–Bonferroni test to identify differences between age groups. The analysis showed differences in all items between groups 1 and 3, whereas it showed no differences between groups 2 and 3. It also identified differences between groups 1 and 2 in the following items: sitting on the floor and getting up, functional reach, standing on one leg on both the left and right sides, obstacle clearance, timed up-and-go, and timed up-and-go with a dual task (Table 4).

## 4. Discussion

The results of this study have confirmed the hypothesis that balance performance was lower in older age groups, suggesting an age-related decline in postural control. The larger differences observed between participants aged 50 and 70 years, compared with those separated by only one decade, likely reflect the cumulative and progressive nature of biological ageing. As impairments in sensory integration, neuromuscular function, muscle strength, and balance accumulate over time, differences in postural control become increasingly apparent across broader age ranges than between consecutive decades.

This study has described postural control behaviour in different age groups, which is useful for the clinical assessment of balance and planning health promotion and rehabilitation interventions. Adults’ balance training represents a protective factor against falls, thereby making it a priority for healthcare teams, especially given the significant comorbidities resulting from falls, i.e., increased length of hospital stays, decreased functionality, and even death [17,18].

Ryckewaert et al. [8] found a strong correlation between FRT performance and the risk of falls in elderly patients and in patients with Parkinson’s disease. The results of this study have highlighted that functional reach performance decreases with age and that the risk of falls increases for those over 70 years old.

A meta-analysis of normative data concerning functional anterior reach by Rosa et al. [19] gave a mean distance of 26.6 cm (25.14: 28.06 95% CI) for adults aged 50–80 years living in a tertiary care facility and 15.4 cm (13.47: 17.42 95% CI) for people not living in care centres. A study by Waroquier-Leroy et al. [9], which included 29 French subjects aged 50 and >75 years, did not find any significant differences in the average weight and height associated with stability performance. There were no statistically significant differences between the groups; however, the FRT score was significantly lower in the 75-year-old group than in the under-50-year-old group (*p* = 0.003), thereby coinciding with this study’s findings and with a decrease in size as age increases.

Dani et Al. [20] in India examined 521 healthy 40–70-year-old adults, finding that this group’s average anterior FRT value was 34.94 ± 3.9 cm. The 51–60-year-old age group’s average performance was 33.99 ± 1.17 cm compared with 28.71 ± 1.05 cm for the 61–70-year-old group. Furthermore, a significant negative correlation was observed between FRT and weight and BMI, thereby showing an age-related decrease for both males and females.

In contrast, a study by Martins et al. [21], which included 20 healthy Brazilians with a mean age of 55.15 ± 3.48 years, found no significant associations between anthropometric parameters and FRT performance.

Springer et al. [17] and Bohannon et al. [18] found that the ability to maintain balance on one leg began to decrease significantly from the age of 60 years and was not related to gender. Furthermore, they highlighted the great reliability of this test among evaluators, thereby supporting its use in clinical practice.

Sgaravatti et al. [22] emphasised that the walking speed (WS) of adults aged 50–80 years decreased with age and was related to a loss of independence regarding daily activities and a higher risk of falls; a mean WS of 1.16 m/s was observed in their sample of Uruguayan people. Furthermore, the average WS in a similar study conducted in Colombia was 0.94 m/s for walking on a stable surface (over 6 m), which was lower than that in Sgaravatti’s study, perhaps influenced by the age range considered in Colombia [23].

Isles et Al. [24] reported that balancing skills began to noticeably decline from age 40 onwards. The study confirmed a decrease in mediolateral stability by the age of 60 years and emphasised the importance of clinical tests for detecting such decline early on. The results highlighted the relevance of adapting tests to individual characteristics, such as height, and suggested the need for targeted interventions (i.e., physical activity) to minimise the risk of falls.

Ansai et al. [25] used a sample of 118 participants to identify the risk of falls in adults aged 50–80 years; 40 of them had preserved cognitive function, with an average age of 73.5 years (SD: 6.2). The results showed that the timed up-and-go test (TUG) performance outcome was 13.1 s (SD: 6.0), which was greater than that found in this study, i.e., 9.3 s (SD: 1.8) for all participants.

Differences in support time between the right (10.3 s) and left legs (9.8 s) suggested dominance regarding postural control. Furthermore, the longer time taken to walk 3 m than 6 m could have been related to dual tasks and postural adjustments during task execution.

The present study found a significant correlation between FRT scores and stature, weight, and age (Table 5), thereby coinciding with the findings of Zawadka et al. [26].

## 5. Conclusions

Balance performance was lower in older age groups, suggesting an age-related decline in postural control, thereby increasing the risk of falls. Thus, beginning proprioception, flexibility, and strength exercises from the age of 40 is indispensable to prevent falls and functional decline.

This study has described the postural control behaviour of adults, identifying the deterioration in such ability during each decade. This information enables the assessment of the balance of older adults and the planning of preventive and rehabilitation actions where training is a priority due to the comorbidities associated with falls. This study confirmed that balance and walking speed significantly decrease with age, thereby affecting functional independence. Future studies should consider the dominance and performance of dual-task activities. To promote healthy ageing, a proven self-care strategy requires participating in exercise programmes before the age of 40.

The importance of addressing balance from a public health perspective lies in the need to implement strategies that promote active and healthy ageing. Such strategies should include intersectoral policies that focus on preventing chronic diseases, promoting healthy lifestyles, and strengthening social support networks. These actions are fundamental for mitigating the impact of demographic transition on health systems and ensuring a better quality of life for the older population while contributing to the country’s sustainable development.

## 6. Limitations

This study has certain limitations that should be acknowledged: the cross-sectional design, convenience sampling, predominance of female participants, absence of longitudinal follow-up, potential influence of physical activity levels, comorbidities, medication use, and fear of falling, as well as the use of selected functional balance tasks rather than a comprehensive multidimensional balance assessment, where applicable.

## Figures and Tables

**Table 1:** Age group descriptors.

	Total	Group 1 *	Group 2 *	Group 3 *	Chi^2^
	*n* = 211	*n* = 81	*n* = 73	*n* = 57	
		*n* (%)	*n* (%)	*n* (%)	*p*-Value
Gender					
Male	68 (32.2)	29 (35.8)	23 (31.5)	16 (28.1)	0.624
Female	143 (67.8)	52 (64.2)	50 (68.5)	41 (71.3)	
Clinical state †					
Healthy	145 (68.7)	54 (66.7)	54 (73.9)	37 (64.9)	0.205
Neurological	12 (5.7)	2 (2.5)	4 (5.5)	6 (10.5)	
Musculoskeletal	54 (25.6)	25 (30.8)	15 (20.6)	14 (24.6)	
	**Total**	**Group 1**	**Group 2**	**Group 3**	**Kruskal–Wallis**
BMI *					
Mean	28.2	28.5	28.1	27.7	0.4894
SD	4.4	4.8	4.1	4.2	
Median	27.4	27.8	27.5	26.7	
Interval (min-max)	18.3–44.6	18.7–44.6	18.3–39.1	18.5–44.5	
Stature **					0.0002
Mean	1.57	1.60	1.57	1.53	
SD	0.09	0.08	0.10	0.09	
Median	1.56	1.59	1.55	1.53	
Interval (min-max)	1.37–1.83	1.38–1.81	1.41–1.83	1.37–1.76	

Source: Own work * Group 1 (≥50 ≤60 years), group 2 (>60 ≤70 years) and group 3 (>70 ≤80 years) BMI: Body mass index (continuous measurement). SD: standard deviation ** Shapiro–Wilk’s *p* value < 0.01. † “Healthy” refers to adults who did not present any clinical state which affected their balance. “Neurological” refers to people suffering sequelae regarding neurological diseases such as Parkinson’s, stroke, multiple sclerosis and/or epilepsy. “Musculoskeletal” refers to people suffering from sequela regarding musculoskeletal diseases such as arthrosis, osteoporosis, scoliosis and/or herniated/slipped discs. Group 1 (50–60-years), group 2 (61–70-years), and group 3 (71–80-years).

**Table 2:** Bivariate analysis by gender.

	Male	Female	Total	Mann–Whitney
	*n* = 68	*n* = 143	*n* = 211	*p*-Value
Sitting on the floor and getting up again (seconds)				
Mean	9.1	10.3	9.9	0.2385
SD	5.8	8.6	7.8	
Median	7.7	8.8	8.1	
Median 95%CI	6.0–8.6	7.6–9.8	7.5–9.0	
Interval (min-max)	2.4–32.0	0.0–75.0	0.0–75.0	
Functional reach (centimetres)				
Mean	27.5	25.0	25.8	0.0242
SD	6.7	7.3	7.2	
Median	27.0	25.0	26.0	
Median 95%CI	25.0–30.0	23.0–26.5	24.0–27.0	
Interval (min-max)	15.0–42.0	3.5–43.0	3.5–43.0	
Standing on one leg—right (seconds)				
Mean	16.2	13.3	14.2	0.0864
SD	12.0	11.4	11.6	
Median	14.2	10.0	10.3	
Median 95%CI	8.5–25.8	6.2–13.4	8.4 –14.3	
Interval (min-max)	0.0–30.0	0.0–30.4	0.0–30.4	
Standing on one leg—left (seconds)				
Mean	15.5	13.1	13.9	0.1056
SD	11.9	11.8	11.8	
Median	12.6	8.4	9.8	
Median 95%CI	7.2–20.0	6.3–11.5	7.4–13.0	
Interval (min-max)	0.0–30.0	0.0–30.0	0.0–30.0	
Stepping up onto a step, alternately (seconds)				
Mean	9.7	9.4	9.5	0.8887
SD	3.3	2.9	3.0	
Median	8.8	9.0	8.9	
Median 95%CI	8.1–9.5	8.5–9.5	8.5–9.4	
Interval (min-max)	5.0–23.2	1.6–18.8	1.6–23.2	
Walking on a stable surface for 6 m (seconds)				
Mean	6.3	7.1	6.9	0.0052
SD	1.6	2.7	2.4	
Median	6.0	6.7	6.4	
Median 95%CI	5.7–6.3	6.4–6.9	6.2–6.7	
Interval/range (min-max)	3.3–11.6	3.6–29.8	3.3–29.8	
Obstacle clearance—6 m (seconds)				
Mean	6.7	7.3	7.1	0.0055
SD	1.7	1.8	1.8	
Median	6.3	7.0	6.8	
Median 95%CI	6.0–6.7	6.8–7.2	6.5–7.1	
Interval (min-max)	4.2–12.0	3.3–13.8	3.3–13.8	
Get-up-and-go—3 m (seconds)				
Mean	9.0	9.4	9.3	0.1498
SD	1.6	1.9	1.8	
Median	8.6	9.1	9.1	
Median 95%CI	8.2–9.4	8.7–9.6	8.7–9.4	
Interval (min-max)	6.6–13.3	5.8–15.1	5.8–15.1	
Get-up-and-go with dual task—3 m (seconds)				
Mean	10.2	10.8	10.6	0.2792
SD	2.5	3.0	2.9	
Median	9.7	10.1	9.9	
Median 95%CI	9.1–10.2	9.6–10.6	9.5–10.3	
Interval (min-max)	6.1–17.1	6.1–23.4	6.1–23.4	

Source: Own work.

**Table 3:** Bivariate analysis by age group.

	Total	Shapiro–Wilk Test	Group 1	Group 2	Group 3	Kruskal–Wallis Test
	*n* = 211	*p*-Value	*n* = 81	*n* = 73	*n* = 57	*p*-Value
Sitting on the floor and getting up again (seconds)						
Mean	9.9	*p* < 0.001	7.3	11.0	12.3	0.0001
SD	7.8		4.2	7.5	10.7	
Median	8.1		6.1	9.3	11.0	
IQR (p25-p75)	5.5–12.7		4.6–8.8	6.1–13.5	6.5–16.0	
Interval (min-max)	0.0–75.0		0.0–25.0	0.0–40.2	0.0–75.0	
Functional reach (centimetres)						
Mean	25.8	0.62362	27.9	25.2	23.4	0.0007
SD	7.2		7.3	6.7	7.0	
Median	26.0		29.0	25.0	23.0	
IQR (p25-p75)	20.0–31.0		22.0–33.0	20.0–30.0	19.0–28.0	
Interval (min-max)	3.5–43.0		3.5–43.0	13.5–41.0	8.0–42.0	
Standing on one leg—right (seconds)						
Mean	14.2	*p* < 0.001	17.9	12.9	10.6	0.0016
SD	11.6		12.0	10.8	10.9	
Median	10.3		19.0	9.7	6.1	
IQR (p25-p75)	3.6–30.0		6.0–30.0	3.7–22.0	2.0–20.0	
Interval (min-max)	0.0–30.4		0.0–3.00	0.0–30.0	0.0–30.4	
Standing on one leg—left (seconds)						
Mean	13.9	*p* < 0.001	18.1	12.0	10.2	0.0003
SD	11.8		12.0	11.3	10.5	
Median	9.8		21.0	7.0	5.6	
IQR (p25-p75)	3.0–30.0		6.4–30.0	3.0–24.0	2.0–17.6	
Interval (min-max)	0.0–30.0		0.0–30.0	0.0–30.0	0.0–30.0	
Stepping up onto a step, alternately (seconds)						
Mean	9.5	*p* < 0.001	8.7	9.5	10.6	0.0012
SD	3.0		2.4	3.2	3.3	
Median	8.9		8.3	8.9	10.1	
IQR (p25-p75)	7.6–11.1		7.1–10.1	7.7–11.0	8.1–13.0	
Interval (min-max)	1.6–23.2		4.8–16.1	2.8–23.2	1.6–18.8	
Walking on a stable surface—6 m (seconds)						
Mean	6.9	*p* < 0.001	6.3	6.8	7.9	0.0038
SD	2.4		1.4	1.5	3.8	
Median	6.4		6.2	6.6	7.1	
IQR (p25-p75)	5.6–7.6		5.3–6.9	5.8–7.4	5.8–9.1	
Interval (min-max)	3.3–29.8		3.6–10.7	4.2–12.2	3.3–29.8	
Clearing obstacles—6 m (seconds)						
Mean	7.1	*p* < 0.001	6.5	7.1	8.0	*p* < 0.001
SD	1.8		1.4	1.5	2.2	
Median	6.8		6.4	6.9	7.8	
IQR (p25-p75)	6.0–8.0		5.5–7.3	6.1–7.8	6.3–9.7	
Interval (min-max)	3.3–13.8		3.3–9.7	3.5–12.2	5.0–13.8	
Timed get-up-and-go—3 m (seconds)						
Mean	9.3	*p* < 0.001	8.6	9.3	10.2	*p* < 0.001
SD	1.8		1.5	1.6	2.2	
Median	9.1		8.4	9.4	10.0	
IQR (p25-p75)	7.9–10.3		7.5–9.7	8.1–10.1	8.4–11.6	
Interval (min-max)	5.8–15.1		5.8–12.5	6.6–13.6	6.9–15.1	
Get-up-and-go with dual task—3 m (seconds)						
Mean	10.6	*p* < 0.001	9.4	10.8	11.9	*p* < 0.001
SD	2.9		2.2	2.9	3.1	
Median	9.9		9.2	10.2	11.4	
IQR (p25-p75)	8.6–12.0		7.9–10.4	9.0–11.7	9.2–14.5	
Interval (min-max)	6.1–23.4		6.1–16.8	7.0–23.4	6.8–20.5	

Source: Own work. SD: standard deviation, min: minimum, max: maximum, IQR: Inter-quartile range, p25: 25th percentile, p75: 75th percentile.

**Table 4:** Dunn–Bonferroni post hoc tests.

	G1 vs. G2		G1 vs. G3		G2 vs. G3	
	z-Dunn’s	*p*-Value	z-Dunn’s	*p*-Value	z-Dunn’s	*p*-Value
Sitting on the floor and getting up again	−3.9	0.0001	−4.3	*p* < 0.001	−0.6	0.8180
Functional reach	2.5	0.0201	3.7	0.0003	1.4	0.2620
Standing on one leg—right	2.3	0.0290	3.5	0.0006	1.3	0.2857
Standing on one leg—left	3.1	0.0033	3.8	0.0003	0.9	0.5792
Stepping up onto a step, alternately	−1.7	0.1369	−3.7	0.0004	−2.1	0.0602
Walking on a stable surface	−2.0	0.0648	−3.3	0.0016	−1.4	0.2591
Clearing obstacles	−2.3	0.0352	−4.2	*p* < 0.001	−2.1	0.0589
Timed get-up-and-go	−2.6	0.0141	−4.6	*p* < 0.001	−2.1	0.0512
Get-up-and-go with dual task	−3.2	0.0018	−5.0	*p* < 0.001	−1.9	0.0770

Source: own work. G1: group 1 (≥50 and ≤60-years), G2: group 2 (>60 and ≤70-years), G3: group 3 (>70 and ≤80-years).

**Table 5:** Spearman’s correlation coefficient.

	Stature	Weight	BMI	Age
	(*p*)	(*p*)	(*p*)	(*p*)
Sitting on the floor and getting up	−0.10	−0.07	0.00	0.35 **
Functional reach (in cm)	0.36 **	0.18 *	−0.06	−0.27 **
Standing on one leg—right	0.20 **	0.05	−0.07	−0.25 **
Standing on one leg—left	0.22 **	−0.01	−0.16 *	−0.30 **
Stepping up onto a step, alternatively	−0.13	0.01	0.13	0.30 **
Walking on a stable surface	−0.25 **	−0.06	0.10	0.24 **
Clearing obstacles	−0.30 **	−0.11	0.11	0.29 **
T imed get up and go	−0.23 **	−0.01	0.16 *	0.33 **
Get up and go with dual task	−0.24 **	−0.14 *	0.01	0.38 **

* *p* value < 0.05; ** *p* value < 0.01.

## Data Availability

The data presented in this study are available upon request from the corresponding author, as the participants did not consent to the public sharing of their data.

## Abbreviations

The following abbreviations are used in this manuscript:

BMI	Body mass index
FRT	Forward functional reach test
TUG	Timed up-and-go
WS	Walking speed
BESTest	Balance evaluation systems test
WHO	World Health Organization
DANE	Departamento Administrativo Nacional de Estadística
